# How to Communicate Food Safety after Radiological Contamination: The Effectiveness of Numerical and Narrative News Messages

**DOI:** 10.3390/ijerph17124189

**Published:** 2020-06-12

**Authors:** Hanna Valerie Wolf, Tanja Perko, Peter Thijssen

**Affiliations:** 1 Fellow at Research Foundation Flanders (FWO)/Department of Political Science, University of Antwerp, 2000 Antwerp, Belgium; 2 Institute for Environment, Health and Safety, SCK•CEN Belgian Nuclear Research Centre, 2400 Mol, Belgium; tanja.perko@sckcen.be; 3 Department of Political Science, University of Antwerp, 2000 Antwerp, Belgium; peter.thijssen@uantwerpen.be

**Keywords:** risk communication, food safety, environmental communication, media effects, nuclear energy, message effects, food risk, radiological contamination

## Abstract

Food risk and the safety of foodstuffs in the aftermath of contamination are highly sensitive issues to communicate. Food risks receive extensive attention from the news media, which requires messages to be carefully drafted to minimize harm and avoid unnecessary boycotts. Once a food risk is deemed eliminated, communication efforts must rebuild trust among consumers. The latter is a particularly difficult task after radiological contamination. This study tests whether numerical messages, narrative messages, or messages combining both elements are more effective in persuading the public to consume foodstuffs from Fukushima, a region that continues to battle stigma since the nuclear accident in 2011. We employ a survey-embedded experiment on a sample of the general Belgian population (*N* = 1085), during which respondents are presented with a mock news article presenting either a (1) numerical, (2) narrative, or (3) a combined message and test their subsequent evaluation of the article. We find that the numerical message leads to significantly higher perceived credibility and message acceptance than both the combined and the narrative message. Furthermore, we find that attitudes towards nuclear energy have a strong independent effect on message acceptance and evaluation, with those respondents who report a more positive stance towards nuclear energy more readily accepting the message. Food risk and safety communication may thus benefit from adopting a more detached, numerical approach for sensitive issues.

## 1. Introduction

Food-related issues are a great concern for science and consumers [1,2]. Evidence suggests that food risks are perceived differently from non-food risks [3], on the one hand because a complete avoidance of food risks is not possible, on the other hand because food has cultural, symbolic, and religious connotations [4]. The human relationship with food is intimate, particularly in an era where the food market is increasingly used as a political arena [5]. Communicating risks associated with food is difficult, even more so when this risk is connected to radioactivity. In such instances, risk communication faces challenges and poor execution may lead to a prolonged boycott of foodstuffs from a country that has suffered a radiological accident [6,7], a rejection of irradiated food by consumers [8], or an unawareness of the natural radioactivity of food among the public [9].

Links between radiation and food products have a negative image among the public, yet radiation is linked to food in different ways, not all of which are harmful to consumers. For instance, many consumers are unaware that radioactivity occurs naturally in food, notably in bananas, carrots, and beer. While natural levels are usually low and safe to consume, they vary depending on factors such as local geology, climate and agricultural practices, and may under certain circumstances exceed the level of radioactivity detectable in products from regions that have suffered a nuclear accident. Risk communication on this issue requires a balance between informing the public about natural radioactivity, while attenuating the negativity connected with radioactivity to prevent a rejection of foodstuffs.

As with naturally occurring radioactivity, the public is widely unaware of food irradiation, a man-made preservation method that involves destroying pathogenic organisms, including *Salmonella* and *E. Coli*, by exposing food to radiation in order to increase its safety. Food irradiation is endorsed by the World Health Organization (WHO), yet despite its benefits, people hold negative views about it [10,11] and many producers are reluctant to indicate irradiation in their products [12], even if all food containing irradiated ingredients must be labeled as such [13]. Every second adult in Belgium is unaware of the use of ionizing radiation [13] and three out of four wrongly think food irradiation makes food radioactive [10], which indicates another challenge for food safety communication.

Man-made radiation can thus be used to increase food safety, but it may also present health risks when food is contaminated with radionuclides above legal norms, for instance after a nuclear accident. While irradiation does not induce radioactivity in food, an accidental release of radionuclides into the environment may, and therefore requires immediate communication to prevent health-related consequences. Such communication may have an effect on the public’s risk perception as research in Belgium has shown, where the effect of different information sources was tested in the aftermath of the Fukushima accident [14]. But effective food risk and post-emergency food safety communication around this issue present difficult tasks for health communication, not least because public outrage around nuclear risk is severe, which can, according to Ju and colleagues [15], lead to an abundance of news stories and panic. While abounding media attention may be fruitful to reach a large audience, messages can become less controllable and vulnerable to misinformation. Severe public outrage may furthermore hinder effective communication of recovered food safety in the aftermath of a nuclear accident. It is thus important to investigate which messages may be most effective in inducing the desired changes in public opinion and behavior for delicate matters such as food risk.

Potential radioactive food contamination as a consequence of nuclear accidents is a primary concern for emergency management [16]. The Chernobyl and Fukushima nuclear accidents demonstrated that food risk communication significantly impacts the management of nuclear disasters and that the stress nuclear accidents place on food systems can threaten human welfare and even nearby survival [6]. People’s risk perception of radiological contamination is extremely high in relation to food supply [17]. Research shows that consumers may avoid consuming products from affected areas in the aftermath of an accident until years after the accident, despite safety guarantees issued by authorities [6,16,18]. This occurs even when products are contaminated below legal norms and the level of contamination is similar to naturally occurring radiation. Thus, food products which are perfectly safe to consume may be rejected by consumers [19]. For regions affected by nuclear accidents, this means further negative consequences beyond the immediate effects of an accident.

Effective risk communication forms the basis of successful food safety and emergency management. A content analysis of newspapers in Europe and Russia in the aftermath of Fukushima examined the use of measurement units and risk comparisons in news articles, as well as the quality of statements on risk related issues including food safety [20]. The authors suggest that risk communicators should improve their practices by presenting information related to (food) safety and risk in several ways, presenting both the results of the radioactivity measurements (numerical presentation) and benchmarks to compare those levels to (narrative presentation) to avoid miscommunication towards consumers, which occurred after the Fukushima accident [20]. Related studies in other branches of communication research, in particular health communication [21,22], have also investigated the persuasiveness of different message types and found that people may react strikingly different depending on whether they receive more detached numerical or more subjective personal testimonies.

This article applies these findings to the issue of food risk communication after nuclear contamination. It addresses a gap in risk communication by identifying whether numerical messages, narrative messages, or messages combining both elements are more effective in persuading consumers to make a seemingly counter-intuitive decision, namely to consume food from a region that has suffered a radiological accident. Drawing on a theoretical framework rooted in exemplification and evidence effectiveness theories, we investigate to what extent these different types of messages affect consumers’ perceived risk and satisfaction with the given information. This study offers valuable insights for environmental risk and food safety communication as well as for emergency management in the aftermath of potential radiological accidents.

### 1.1. The Use of Numerical and Narrative Evidence in Persuasive Communication

Communication about food issues requires carefully drafted messages with convincing evidence if it is to persuade the public to adapt its perceptions and/or behaviors, particularly if there is an incongruence between its instinctive and informed decision making. From persuasive communication research, we know that different evidence types may result in considerably different responses. So-called ‘evidence’ is needed to substantiate an argumentative claim in order to persuade a recipient that a claim is indeed true, and, for instance, in the domain of health or risk communication, to advocate a change in behavior. Evidence as such is central to any (effective) communication and can be defined as “data (facts or opinions) presented as proof for an assertion” [23] (p. 429).

Evidence effectiveness literature commonly applies a dyadic differentiation between numerical and narrative evidence [24], with some scholars differentiating more narrowly between statistical, anecdotal, causal [25], and sometimes expert evidence [26]. A recent article by Wojcieszak and Kim [24] defines numerical messages as “arguments that utilize numbers to advance a point of view” (p. 787), which entails “empirically quantified descriptions of events, persons, places, or other phenomena” [27] (p. 108). Narrative messages have been defined as representations of “events and characters, with an identifiable structure” [24] (p. 787), testimonials [28], but also as vivid descriptions and metaphors, which departs from the notion of evidence and focuses on the stylistic characteristics of the message type. Since the operationalization of evidence types differs considerably between researchers, authors unsurprisingly note varying effects [25,29]. The mechanism of contrasting and comparing the effects of different message types is closely linked to the theory of exemplification, a well-established communication theory that uses experimental methods to document how different messages are cognitively processed and retrieved by humans in order to make both attitudinal and behavioral judgements [30]. The branch of evidence effectiveness research largely draws on this theory and its methodology of contrasting messages and merges it with classic theories on the use and hierarchy of evidence types in argumentation. Evidence effectiveness research is therefore predominantly focused on the perspective (perceived subjectivity/objectivity) and the resulting (perceived) validity of an evidence type, whereas exemplification theory in its original form is more concerned with the difference between emotionally arousing ‘exemplars’ and abstract, inconsequential representations.

Our focus in this paper is a comparison between a numerical message that presents evidence in the form of a normed statistic, a narrative message in the form of a personal testimony, and a combination of the two types above, which reflects the fact that news articles commonly make use of both evidence types within an article, which has often been neglected in prior research even though a number of scholars have voiced a call for its inclusion. We believe the operationalization of our two core evidence types, on the one hand, provides a clear distinction between them and, on the other hand, realistically reflects how journalists use the two types of evidence when reporting risk in real news articles. As indicated above, there are numerous ways in which both numerical and narrative messages can be operationalized. Our study aims to compare the effects of two typical representations, while acknowledging that effects may differ for less pronounced examples of, for instance, narrative messages (e.g., a subtle change in the vividness of a message).

When communicating risk to the general public in a numerical manner, journalists are instructed to not only report the (change in) risk itself, but also the corresponding baseline figure in order for the recipient to make sense of the number’s significance. Several broadcasting houses and news organizations have formulated explicit editorial guidelines on the correct reporting of numbers, and on reporting risk specifically. BBC News, for instance, state that “where there is editorial justification for reporting changes in risk, it would be meaningless if our reports did not include the baseline risk” [31]. We thus opt to operationalize our numerical message in the form of a normed statistic in our experiment, which is less dependent on a subjective interpretation on the side of the recipient than, for example, a percentage, and at the same time reflects real-life, responsible journalism.

We chose a personal testimony as the narrative counterpart to the numerical message, since our focus in this study is to test for the effect of different evidence types (i.e., types of ‘proof’ provided by journalists for a certain claim, whether in the form of numbers or personal accounts), and not the effects of varying emotionality levels in terms of the wording used. A personal testimony adds a profound narrative element into the storyline, which may “increase the ease with which message recipients can imagine an event or construct a scenario” [28] (p. 111). It provides clear, if personal, evidence for the claim in question, thereby making it a distinctive counterpart to the numerical message. While we do not explicitly vary the level of emotionality in our experiment, the inclusion of a personal testimony nonetheless implies a shift towards an individual perspective, which some recipients might perceive as more subjective and self-interested, while others will see it as more lifelike.

### 1.2. The Persuasive Effects of Numerical and Narrative Messages

Despite the aforementioned differences in terms of operationalization, both numerical and narrative messages have been found to exert manifold effects on message recipients. The results regarding which evidence type is superior in effectiveness have remained inconclusive, with some authors stating that narrative evidence is more persuasive [32], while others find more persuasiveness in numerical evidence [33]. Authors have pointed out various explanations for the effects they found, ranging from individual characteristics [34], the level of vividness in a message [35] to the length of an article or the amount of evidence included in a stimulus [36]. Despite the difficulty to answer the question of superiority conclusively, several interesting patterns in response to the different messages have been observed.

Firstly, in terms of message evaluations and acceptance, several studies conclude that numerical messages are rated as more persuasive [29,33,35,37,38], credible and verifiable [39], believable [21], of higher writing quality [34] as well as of higher informational value [22] than narrative messages. Some authors note that people prefer narrative evidence on relatively personal issues [40,41,42], whereas numerical information is perceived as more useful for abstract and complex topics, as well as for distant threats such as climate change [42]. A message providing both numerical and narrative elements may, however, be the most powerful message, as it satisfies the consumers’ demand for credibility, but also allows them to relate to the message on a more personal level. Communication on food risk related to radiological contamination hinges on being perceived as credible by the public, but it must overcome deep-rooted perceptions of negativity surrounding radioactivity. Since radiological accidents are nonetheless rare and not perceived as an imminent threat, we expect that

**Hypothesis** **1.** *Numerical messages will be perceived as more credible than narrative messages.*

**Hypothesis** **2.** *Numerical messages will receive higher levels of message acceptance than narrative messages.*

**Hypothesis** **3.** *Messages combining numerical and narrative elements will receive the highest credibility and acceptance ratings.*

Besides positive message evaluations, a number of authors observe another interesting pattern. [43] find that numerical messages lead to more cognitive responses than narrative messages and argue that this difference might be “a result of difficulty in disputing someone’s story” (p. 296) as opposed to disputing more objective, numerical evidence. Research by Slater and Rouner [34], too, finds considerable differences in the effects of numerical and narrative evidence for different groups of recipients, namely that value-affirmative recipients (who process value-congruent messages as a means to reinforce existing beliefs) use numerical messages to reinforce existing beliefs, while value-protective recipients (who defend their values against value-discrepant messages) tend to counter-argue against numerical evidence. Defensive strategies when presented with numerical evidence have also been noted by Thorne et al. [44], who interviewed cancer patients and found that those patients who were philosophically or bio-medically skeptical tended to discount numerical information more often and came to disregard it as ”only numbers” (p. 326). Such defensive strategies in personal risk assessment relate strongly to the findings of established research on self–other discrepancies in risk perception, where individuals assess their own risk as substantially lower than that of the general public [45]. This mechanism can be broadened to the differential effects of (objective) numerical messages and individual testimonies that present a claim with a potential risk to the message recipients. Since a personal testimony commonly describes what is at stake for this specific individual, recipients may be inclined to relate to said personal stakes of the individual more deeply than to the objective presentation in terms of numbers. The studies cited above suggest that personal experiences are more difficult to discount in their entirety; numerical messages may thus more readily be perceived as biased than narrative messages when it comes to the assessment of a counter-intuitive claim.

Besides induced defensiveness when presented with numerical evidence, what has often been neglected is the effect messages combining personal experiences and supporting numerical evidence have on bias perceptions. Combined messages present more robust evidence [20], since a personal (subjective) testimony that recipients may find harder to dismiss as biased is corroborated by (objective) numbers. Even for recipients who find a claim counter intuitive, a message providing two sources of evidence that appeal to both empathic relatability and what one might call “hard facts” will be harder to discount than a message providing only one type of evidence. We believe that when numerical and narrative messages are combined and congruent in their assertion, numbers will strengthen and lend broader credibility to a personal account and thus impede the emergence of defensive and dismissive reactions. We therefore expect that

**Hypothesis** **4a.** *Numerical messages will be perceived as more biased than narrative messages.*

**Hypothesis** **4b.** *Combined messages will be perceived as the least biased messages.*

### 1.3. Individual Predispositions: Different Consumers, Different Preferences?

We must also consider that besides regular socio-demographic differences in age, sex and education, further variables may influence the evaluation of different message types and should, therefore, be controlled for in our analysis in order to establish the robustness of any potential evidence effects. Such variables concern above all individual predispositions and prior issue attitudes, which have previously been found to influence message effects [34]. Prior research has identified several important control variables related to the effectiveness of numerical and narrative messages. Among these are the numerical capacities of a person and, relatedly, a personal predisposition toward numbers, which describes a measure of a person’s comfort in dealing with numbers. A recent study in political communication [24] finds that highly numerate people perceive numerical evidence as persuasive enough to overcome party cue effects. They observed that when a message by an opposing party included numerical evidence, highly numerate individuals accepted it despite a predisposition against the party—a result that was found to be reverse for people with low numeracy skills, who tended to exhibit defensive strategies. While predispositions towards numbers have been identified as relevant for both cognitive and affective processing of numerical information [46,47], conversely, empathic predispositions appear to have the opposite effect: Highly empathic individuals seem to be less susceptible to numerical evidence [24,47], and are instead more favorable towards narrative messages.

In order to retrieve a more complete picture of the dynamics at play in the assessment of different evidence types, we thus include measures for both numerical and empathic predisposition, as well as for the respondents’ attitudes towards nuclear energy and their trust in authorities as control variables in our analyses.

## 2. Materials and Methods

Our dataset derives from a between subjects, post-test only, survey-embedded experiment with three message type conditions (narrative/numerical/combined). The experiment is designed in the form of split-ballot testing—experiments within standardized representative surveys, where the sample is divided into two or more subsamples (conditions) for a limited time, during which each subsample receives a different experimental manipulation at the same point in the survey procedure. The results of the subsamples are then compared against each other rather than against a control group [48]. This is appropriate as our study explores differences between the three message conditions, rather than between an experimental and a control group. As an additional validity check, we nonetheless include a control group which is not exposed to any experimental manipulation. The study forms part of a larger public opinion tracking, a survey which is designed to cover a range of topics related to nuclear energy and is not specific to risk from food. Questions concern, inter alia, the perception of radiological risks, confidence in authorities, opinion about nuclear energy, trust in nuclear actors, knowledge about the nuclear domain, expected behavior in emergency situations and public information issues in nuclear emergencies. This will be conducted upon Belgian adults (SCK•CEN Barometer) to assess public opinion on nuclear energy and the associated risks. The survey was first carried out in 2002 and has been executed by Ipsos Belgium, a company which has specialized in opinion research since 2013. Our data derives from the 2017 edition and was collected between 27 November 2017 and 26 February 2018 by professional interviewers (*N* = 77), who administered face-to-face CAP Interviews at the homes of the respondents. On average, an interviewer conducted 14.1 interviews. In order to reduce the overrepresentation of easy-to-reach respondents, 20% of all interviews were conducted on weekends and an additional 20% of interviews were conducted after 5 pm on weekdays.

Respondents in the sample were selected using a random walk approach and a stratified sampling methodology to select Primary Sampling Units (Communes). The sampling frame was stratified according to urbanization degree (three strata) and province (11 strata). For each sampling point, one address was drawn at random. From this address, a cluster of households was formed by selecting every third household by standard random route procedure.

For our study, it was particularly important that the questionnaire was administered face-to-face because the reliable execution of our survey-embedded experiment relies heavily on the respondents’ careful reading of the news story we present them with. A different method would not allow comparable control over the correct execution of the experiment. All subjects gave their informed consent for inclusion before they participated in the study. The study was conducted in accordance with the Declaration of Helsinki, and the protocol was approved by a steering committee of experts in the field of survey design and survey ethics (Members of the steering committee: 1. Prof. Britt-Marie Drottz Sjøberg, Norwegian University for Science and Technology, Trondheim, Norway, 2. Prof. Jaak Billiet, KU Leuven, Belgium, 3. Prof. Richard Eiser, University of Sheffield, U.K., 4. Prof. Peter Thijssen, University of Antwerp, Belgium, 5. Dr. Frank Hardeman, director of Nuclear Safety Authority FANC-AFCN, Belgium, 6. Ir. Geert Volckaert, FANC-AFCN, Belgium).

Our sample consists of *N* = 1085 Belgian adults and is representative of the (18+) Belgian population with respect to gender, age, education, and the level of urbanization. The respondents in the experimental conditions were randomly assigned to either a numerical (*N* = 220), a narrative (*N* = 200) or a combined stimulus (*N* = 198) on the issue of post-emergency safety of foodstuffs from Fukushima by means of a computerized randomization procedure and resulted in no significant socio-demographic differences between the conditions. The control group (*N* = 467) was not exposed to any information about this issue. For the survey, our experiment involved several research teams and two planned experiments. In order to maximize data quality and to minimize respondent refusal, the decision was taken to split the total sample into two groups for the administration of the two experiments (subsample 1: *N* = 618, subsample 2: *N* = 467), resulting in two parallel experiments. For increased statistical power, the respective other subsample served as a control group. For this study, participants in subsample 1 were randomly assigned to one of three experimental conditions and respondents in subsample 2 (no stimulus) received a number of control questions. The parallel experiment was similar in length and design, but unrelated to food risk. The survey resulted in a response rate of 7% (summary of outcome rates: *Response rate*: Proportion of all eligible respondents that resulted in a completed interview: 7%. *Cooperation rate*: Proportion of all eligible respondents contacted that resulted in a completed interview: 17%. *Refusal rate*: Proportion of all eligible respondents that refused to participate: 29%. *Contact rate*: Proportion of all eligible respondents that were contacted: 42%). A pilot study with 25 respondents was carried out as a pre-test of the survey. To ensure the fieldwork was conducted uniformly, back-checks were carried out by telephone on a total of 234 completed interviews.

### 2.1. Stimulus Materials

**Figure A1:**
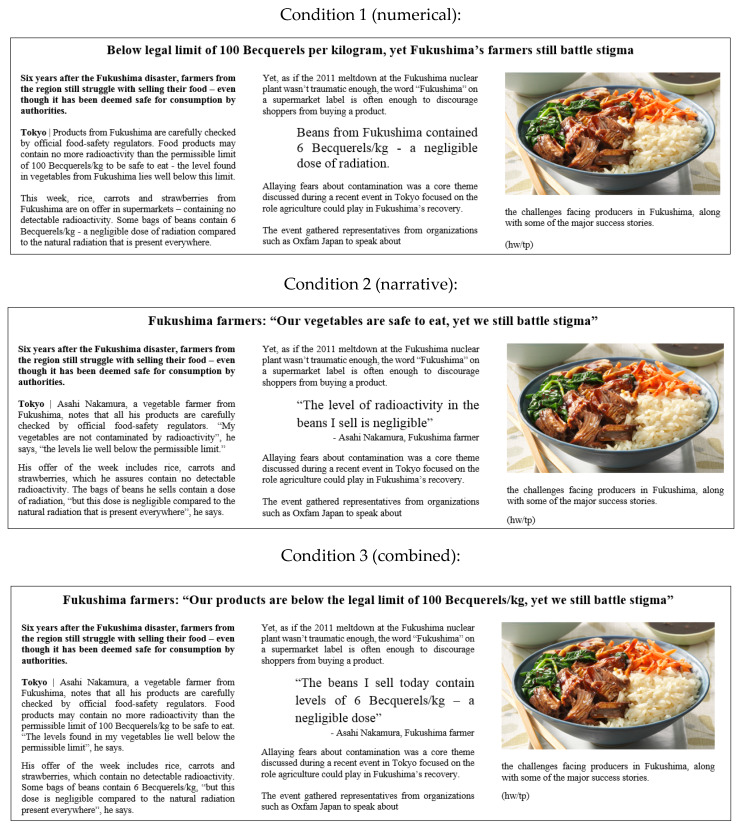
Experimental stimuli (translated from Dutch to English).

The stimuli (Figure A1, Appendix A) were presented as newspaper articles on food from Fukushima. All respondents assigned to one of the three experimental conditions read the same news story, wherein certain key elements were manipulated according to the respective condition they were in, such as the headline and pull quotes in the article. The format of newspaper articles was selected since people most frequently use mass media when they seek information about food safety [2] and because Belgian newspapers followed the Fukushima accident closely.

Respondents were randomly assigned to one of the three experimental conditions. Respondents read one out of three mock news articles on products from Fukushima, which are safe to consume but struggle to sell. The stimuli thus contain a counter-intuitive appeal, namely, to consume produce from Fukushima. The numerical stimulus expresses this claim in terms of absolute numbers, e.g., “Below legal limit of 100 Becquerel per kilogram, yet Fukushima’s farmers still battle stigma”, whereas the narrative stimulus makes use of a personal testimony including direct quotations, e.g., “Fukushima farmers: Our vegetables are safe to eat, yet we still battle stigma”. The combined stimulus uses both types of evidence: the personal testimony (vegetable farmer) and the provision of numbers (100 Becquerel/kg), e.g., “Fukushima farmers: Our products are below legal limit of 100 Becquerel per kilogram, yet we still battle stigma”. In this latter stimulus, the same vegetable farmer as in the narrative condition presents the story from his perspective, but additionally provides the numbers used in the stand-alone numerical condition. Since this study tests for the effect of different evidence types (i.e., only the “proof” presented for a claim), the changes made to the stimuli had to be comparatively subtle to avoid the introduction of confounding variables, such as the tone and length of an article, which have previously been found to influence the effectiveness of different evidence types (e.g., [24]). To ensure comparability of the conditions, the stimuli were kept equivalent in terms of valence, argument and storyline, and kept as similar as possible in terms of length and wording. The chosen issue pertains to a real-world uncertainty in the domain of food risk communication and is in line with post-emergency recovery management procedures. The sample is 52% female with a median age of 41 years old. The modal education level is a higher non-university diploma.

### 2.2. Measurements

#### 2.2.1. Dependent Variables

(a) Message credibility: Measured by means of a 7-point semantic differential adjective scale (‘credible—not credible’). Higher scores indicate higher lack of credibility (*Mean*: 3.83, *SD*: 1.65).
(b) Message bias: Measured by means of a 7-point semantic differential adjective scale (‘neutral—biased’). Higher scores indicate higher perceived bias (*Mean*: 3.97, *SD*: 1.66).
(c) Message acceptance: Measured by means of a 5-point scale item taken from Allen and colleagues’ [49] message acceptance scale (“My opinion is consistent with the argument expressed in the article”). Higher scores indicate higher acceptance levels (*Mean*: 3.19, *SD*: 1.14).

Besides these core dependent variables, which offer insight into the differences between the experimental groups, we furthermore include the dependent variable “risk perception” to test for differences in the article reception between the control group (i.e., no exposure to communication) and the experimental groups (i.e., exposure to one of three experimental conditions). Unlike the previous dependent variables, this variable does not depend on prior exposure to a news story.

(d) Risk perception: Measured by means of two 5-point scale agreement items asking “If I needed a specific ingredient, I would buy it even if it was produced in Fukushima” (*Mean*: 2.33, *SD*: 1.26) and “I would feel uncomfortable eating food produced in Fukushima” (*Mean*: 3.66, *SD*: 1.17). Higher scores indicate stronger agreement with the statement.

#### 2.2.2. Control Variables

(a) Preference for numerical information (PNI): Measured by six 5-point scale items modelled on items from Viswanathan’s [50] PNI scale, including ‘I enjoy work that requires the use of numbers’. Higher scores indicate higher preference for numerical information. The scale resulted in a highly reliable Cronbach’s alpha of 0.88 (*Mean*: 3.16, *SD*: 0.99).
(b) Empathic concern: Measured by four 5-point scale items taken from Davis’s [51] empathic concern items, including ‘I am often quite touched by things that I see happen’. Higher scores indicate higher reported empathic concern. The scale resulted in a reliable Cronbach’s alpha of 0.76 (*Mean*: 4.00, *SD*: 0.75).
(c) Attitudes towards nuclear energy: Adapted from the GlobeScan survey on global nuclear energy attitudes [52] and measured by six 5-point scale items, including ‘The reduction of the number of nuclear power plants in Belgium is a good thing’. Higher scores indicate more positive attitudes towards nuclear energy. The scale resulted in an acceptable Cronbach’s alpha of 0.73 (*Mean*: 2.67, *SD*: 0.82).
(d) Trust in authorities: Modelled on the OECD’s institutional trust scale [53] and measured by means of a 5-point scale item asking “How much confidence do you have in the authorities regarding the actions they undertake to protect the population against risks from residues of radioactivity in food?”. Answers ranged from ‘very little’ to ‘very much’. Higher scores indicate higher trust in authorities (*Mean*: 2.95, *SD*: 1.00).

## 3. Results

Preceding the main part of our data analysis, we tested for relevant correlations and mean differences between demographic groups with regards to the control variables. Our data reveal striking differences between the demographic strata in our sample: Women report statistically significant higher levels of empathic concern than men (t (617) = −7.95, *p* < 0.001). Men, on the other hand, show a higher PNI score than women (t (593) = 5.68, *p* < 0.001) and are more likely to hold favorable attitudes towards nuclear energy (t (552) = 3.01, *p* < 0.01). Besides gender, age and the level of education also show interesting patterns in our sample: The younger a person, the higher their PNI (*N* = 593, r = 0.137, *p* < 0.01). In return, the older a person, the higher empathic concern is reported (*N* = 617, r = −0.251, *p* < 0.001). With regards to education, the data show that higher education correlates positively with higher PNI (*N* = 593, r = 0.215, *p* < 0.001) as well as less favorable attitudes towards nuclear energy (*N* = 552, r = −0.112, *p* < 0.01). It is important to note that empathic concern and PNI are also correlated: The higher the PNI, the less empathic concern is reported (*N* = 592, r = −0.122, *p* < 0.01). These findings, while striking, may not be fully generalizable to the general population, since full representativeness of our sample cannot be guaranteed.

To ensure the core message of the article was received correctly, immediately after having read the news story, respondents across the three experimental conditions (*N* = 618) were asked whether, according to the article, food products from Fukushima mentioned in the article were safe for consumption. In total, 77.7% of respondents correctly recalled the message of the article, with no significant differences in the level of correct recall between the three experimental groups. Moreover, a comparison of mean risk perception scores between the experimental groups that were exposed to a message and the control group shows significantly lower scores for those respondents who read a news story (Table A1, Appendix A). We thus observe that irrespective of the message type employed, communication is effective in lowering the perceived risk from a food contamination.

We then proceeded to our main analysis which sets out to test for differential effects of the experimental stimuli by means of a series of linear regression analyses (Table 1, Table 2 and Table 3). To this end, two dummy variables were created to represent the different subgroups of our independent variable evidence type: one for the narrative and one for the combined condition, respectively. The numerical condition acts as the comparison group in the regression analysis. The three models develop as follows: Model 1 controls only for core demographics, Model 2 adds empathic and numerical predispositions, and Model 3 further includes the control variables prior attitudes towards nuclear energy and trust in authorities. Cohen’s d effect sizes per dependent variable and group comparison are reported in Table 4. While our random assignment into the treatment conditions should ensure that differences between the socio-demographic composition of the groups are minimized, we conducted an additional Propensity Score Matching analysis between the treatment (stimuli), *N* = 618, and control (no stimulus), *N* = 467, groups to confirm that the overall communication effect is not confounded by socio-demographic covariates (gender, age, education). Our results show propensity scores ranging from 0.39 to 0.72 with no exact matches, but a total of 467 fuzzy matches. The maximum number of cases was successfully matched between control and treatment groups. To ensure that the results reported in the regression analyses between the three treatment groups (following below) are robust, we then repeated an exemplary regression analysis using the pruned sample of fuzzy matched cases (reduced sample size: *N* = 382). The effects for the stimuli reported in the Results section below remain significant, with, for instance, the numerical stimulus resulting in higher message acceptance than the narrative stimulus (β = −0.44, t = −3.20, *p* = 0.002). The results of our three models are displayed in the tables below. For descriptive values per dependent variable by experimental groups, see Table A2 (Appendix A).

### 3.1. Message Evaluations

One of our core dependent variables is message credibility, which we expect to be higher for numerical messages than narrative messages (Hypothesis 1), but highest for the combined message (Hypothesis 3). Our data in Table 1 show a significant main effect for message type, with the numerical stimulus rated more credible than the narrative stimulus throughout all three models (Model 1: *p* < 0.01, Model 2: *p* < 0.05, Model 3: *p* < 0.05). Issue attitudes show a significant main effect on message credibility (*p* < 0.05), with those in favor of nuclear energy rating the message more credible. Our results therefore support Hypothesis 1, even though the predictive value of our model remains low. Our findings lend no support for Hypothesis 3, which predicted that the combined stimulus would be rated most credible. While it is rated as more credible than the narrative message, it ranks behind the numerical message.

In terms of bias perceptions, our results in Table 2 do not support our hypotheses that numerical messages are perceived as more biased than narrative messages (Hypothesis 4a) or that combined messages receive the lowest bias scores (Hypothesis 4b). Our results show little variation between the three messages in terms of perceived bias across all models. Only Model 3 significantly predicts bias perceptions; however, the effect size is negligible and can only be attributed to the control variables introduced. We thus find no support for Hypothesis 4a and Hypothesis 4b. With regards to bias perceptions, we furthermore find that while women rate all messages as more biased than men regardless of the experimental condition (*p* < 0.05), this effect disappears when controlling for attitudinal characteristics. Prior issue attitudes (*p* < 0.05) and trust in authorities (*p* < 0.01) appear as the strongest predictors for perceived message bias with those in favor of nuclear energy as well as those with higher trust levels rating the article less biased.

### 3.2. Message Acceptance

The results in Table 3 show a strong and robust effect for the message type condition across all three models, with respondents who read a numerical message showing higher levels of message acceptance than those who read narrative (Model 3: *p* < 0.001) or combined (Model 3: *p* < 0.05) messages. While it is surprising that the combined message receives lower scores than the numerical message, thus refuting Hypothesis 3, the difference between the numerical and the narrative message is striking. This lends strong support for Hypothesis 2, even though the effect size remains relatively small. Prior attitudes towards nuclear energy also show a strong main effect (*p* < 0.001), with those respondents with more positive issue attitudes reporting higher levels of agreement. Prior attitudes appear to be a strong and consistent predictor for message evaluations regardless of the message type, as does the level of trust in authorities, which also shows a significant effect on message acceptance levels (*p* < 0.001). Contrary to previous findings, however, neither numerical nor empathic predispositions have a significant effect on message evaluations.

## 4. Discussion

Our study aimed to shed light on the effectiveness of different messages in communicating about recovered food safety in the aftermath of (radiological) contamination. To this end, a survey-embedded experiment among Belgian adults was conducted, in which participants were presented with a mock news article containing either a numerical, a narrative, or a combined message, and subsequently reported their message ratings and risk perception. The results of our study show that numerical messages are more effective than narrative or combined messages in terms of eliciting credibility and acceptance in the field of food safety communication after radiological emergencies. These findings are in accordance with prior research on evidence effectiveness [25,29,37,38,39,54]. We furthermore confirm the finding by Peralta et al. [42] that numerical information may be perceived as useful for abstract topics and distant threats, as the case for food risks related to nuclear emergencies. Despite several prior studies observing defensive strategies among consumers who encounter counter-intuitive numerical information [28], we do not find support for our hypothesis that numerical messages are perceived as more biased than narrative messages. Such bias perceptions may hinge strongly on the source of a message and the respondents’ trust in this source, information we omitted from our experimental setting. Indeed, a recent study by Oshita [55] finds that enhanced trust may balance out negative emotions induced by emergency preparedness communication. It is thus not surprising that our study finds trust in authorities to be a key control variable, a finding confirmed in research by Guo, Li and Chen [56], who found trust in the government to be a key predictor for risk perception and anxiety in relation to nuclear radiation. Furthermore, we cannot confirm previous findings that personal predispositions towards numerical information and the reported level of empathic concern significantly influence the response to the different stimuli. Nonetheless, we observe striking correlational differences between females and males, higher and lower educated, and younger and older respondents in terms of their predispositions. We thus believe that such personal preferences may be relevant for other issues.

Our study does not come without limitations. We tested the differential effects of numerical and narrative evidence; however, the observed effects must be interpreted in consideration of the chosen operationalization. We opted for normed statistics and personal testimonies, which presents a rather clear contrast between the stimuli, as the narrative message is presented from a clearly subjective perspective compared to the more objective, numerical message. Our results are not necessarily transferable to more subtle or merely stylistic alterations between the two message types. Notwithstanding, we believe that a clear distinction between evidence types not only serves to find clearer effects, but also aids in bringing operational clarity to the field of evidence effectiveness research which has struggled with manifold definitions, conflating concepts, and as a consequence, inconsistent findings.

The inclusion of a combined evidence stimulus did not prove as fruitful as we hoped. While it appears to be more effective than a merely narrative stimulus, the combined message failed to overcome the numerical message in effectiveness. Despite enhanced robustness, the combined message did not prove to be more effective in suppressing perceptions of bias, which could indicate that combined messages dilute rather than strengthen the persuasiveness of single sources of evidence. However, we believe this is in part due to the difficulty in doing justice to the combined format in this study, which aimed to keep the stimuli as comparable as possible. Messages using both evidence types may require longer articles that give appropriate space to the integration of the two evidence types. We therefore encourage future research to include combined messages when the scope allows for a more detailed integration rather than a juxtaposing of the two evidence types.

A further limitation we must acknowledge is that our dependent variables were measured by means of single items and as such, it is not possible to assess the reliability of these items. Due to the relatively long and demanding survey our experiment was embedded in, we aimed to minimize respondent refusal by limiting our items to core items, which we believe to be reasonably concrete and therefore appropriate to be measured by a single item, even if not conventionally done. While this presents a drawback, two of the three variables in question were measured on a 7-point semantic differential adjective scale, which provides relatively many points of discrimination and is known to reduce acquiescence bias [57]. Given that our sample size is sufficiently large and several studies suggest that single items can be as able to capture a theoretical concept than multi-item scales—even in the case of attitudinal measures [58]—we are confident that the scores reported for our dependent variables are meaningful.

Furthermore, the issue of food consumption from Fukushima could be considered too distant a threat to provoke striking emotional responses, as it is relatively easy for Belgian consumers to circumvent buying food from this region. However, since nuclear accidents of this caliber are an extremely rare occurrence, the selected region nonetheless presents the best option, as Fukushima is still strongly connected with the accident among the Belgian public and the accident has been widely discussed in the news media. In addition, research found that the news media frequently referred to the Chernobyl disaster when reporting on Fukushima and portrayed potential environmental and health consequences as equally serious [59], which adds to the perception of severity of the case in the mind of the public. Moreover, just prior to the data collection period, the European Union further relaxed its ban on food products from Fukushima and lifted test certificate requirements for products from several prefectures (including rice from Fukushima). Products from Fukushima are available in Belgium and consumers may be unaware that they are exposed to them. Seeing that the control group in our study, which did not receive any information about the safety of food products from Fukushima, perceived the risk from such products as significantly higher than those respondents who received information about it, we believe our study shows the importance of clear communication about issues that consumers may find counter-intuitive or have reservations about. Not addressing it may lead to higher (false) risk perceptions. Considering products from Fukushima destined for the Belgian and European market arrive at the port of Antwerp in Belgium and are subsequently checked for safety by national authorities prior to their distribution to the public, the issue in question may even be particularly relevant for Belgian consumers.

Finally, in order to apply our findings to other uncertainties relating to food risk communication, different scenarios should be compared in further studies. It is plausible that the effects found in our study are different when a less distant threat is presented, such as a contamination by *E. coli* or *Salmonella*. Respondents may perceive such cases as immediately relevant for them on a personal level, and lead them to respond differently.

Based on our results, we formulate several suggestions for communication practitioners:

(1) Data from our sample suggests that providing precise, unambiguous numbers to support a communication may enhance a message’s credibility vis-à-vis the public. Few but well-chosen numbers may present the most adequate strategy.
(2) Based on our study, targeting subgroups of the population with different messages may enhance overall persuasion levels. If an assessment of issue attitudes is possible, we suggest intensifying communication to those consumers who are inclined to be critical towards a given issue, whereas those with a more positive stance might need less frequent messages. Furthermore, efforts towards enhancing the public’s trust in authorities should be undertaken, as low levels of trust appeared to inhibit message acceptance in our sample.
(3) Providing numerical data may be more effective for distant rather than imminent threats. This includes risk communication that prepares for nuclear emergencies. If a threat is imminent, such as an acute food risk, we suggest broadening the scope of messages by also providing narrative messages.

## 5. Conclusions

Food safety is everybody’s concern. Consumers are highly skeptical when it comes to food produced in a region known to have suffered a radiological accident, such as Fukushima. Communication about the safety of food products in particular, but also risk communication in general, have a great responsibility in post-emergency recovery. Poor communication can exacerbate (unnecessary) boycotts of products, whereas sound communication can alleviate fears among the public and provide adequate confidence to consume products that are declared safe. By so doing, risk communication supports the socio-economic recovery of a region suffering from consequences after a radiological contamination. This article addressed a gap in risk communication related to the presence of radionuclides in food by identifying whether numerical, narrative, or combined messages are more effective in persuading consumers to make a decision that may appear counter-intuitive to them, namely to consume food from a region that has suffered a radiological accident.

Results show that any form of communication alleviates the perceived risk when compared to a lack of communication thereof. The strongest positive effects are observed after exposing respondents to numerical messages. Messages combining numerical and narrative elements also appear more effective than merely narrative messages; however, we do not find them to be more effective than numerical messages. The expectation that a combination of two evidence types enhances the robustness of a message cannot be confirmed. We see, however, that prior attitudes towards nuclear energy significantly affect the perceived credibility of a message as well as the acceptance of a message. Respondents with more positive issue attitudes reported higher credibility and acceptance of the message. We also find that trust in authorities has a strong effect on message perceptions, which prior studies often neglected to include. We thus recommend further research to include variables measuring trust in a variety of authorities with societal responsibility.

These results provide important insights for the sensitive task of food safety communication and supports radiological emergency communication in the aftermath of potential radiological accidents.

## Figures and Tables

**Table 1:** Effect of message type on perceived message credibility.

Effect of Message Type on Perceived Credibility									
	Model 1			Model 2			Model 3		
Predictor	β	SE	T	β	SE	t	β	SE	t
(Constant)	16.73	7.65	2.19 **	13.16	8.30	1.59	12.72	9.15	1.39
*Message type* *(reference category: Numerical)*									
Narrative (dummy)	0.43	0.16	2.67 **	0.42	0.17	2.54 *	0.40	0.17	2.32 *
Combined (dummy)	0.14	0.16	0.90	0.15	0.17	0.91	0.20	0.18	1.11
*Control variables*									
Gender	0.15	0.13	1.09	0.12	0.15	0.79	0.07	0.15	0.45
Age	−0.01	0.00	−1.70	−0.01	0.00	−1.18	−0.00	−0.01	−0.88
Education	−0.04	0.03	−1.46	−0.05	0.03	−1.71	−0.07	−0.03	−2.25 *
Numerical preference				−0.04	0.07	−0.53	−0.04	0.08	−0.53
Empathic concern				0.11	0.10	1.15	−0.07	0.10	0.67
Attitudes (nucl. energy)							−0.20	0.09	−2.19 *
Trust in authorities							−0.13	0.07	−1.80
	R^2^ = 0.026 F(5, 613) = 3.284 **			R^2^ = 0.032 F(7, 584) = 2.748 **			R^2^ = 0.051 F(9, 510) = 3.076 **		

*Note.* * = *p* < 0.05, ** = *p* < 0.01.

**Table 2:** Effect of message type on perceived message bias.

Effect of Message Type on Perceived Message Bias									
	Model 1			Model 2			Model 3		
Predictor	β	SE	T	β	SE	t	β	SE	t
(Constant)	5.99	7.73	0.77	6.40	8.46	0.76	3.61	9.30	0.39
*Message type* *(reference category: Numerical)*									
Narrative (dummy)	−0.06	0.16	−0.36	−0.09	0.17	−0.55	−0.08	0.18	−0.43
Combined (dummy)	−0.02	0.16	−0.11	−0.04	0.17	−0.23	−0.02	0.18	−0.12
*Control variables*									
Gender	0.29	0.13	2.19 *	0.29	0.15	1.94	0.20	0.16	1.28
Age	−0.00	0.00	−0.34	0.00	0.00	−0.32	0.00	0.01	0.18
Education	0.04	0.03	1.37	0.04	0.03	1.23	0.01	−0.03	−0.20
Numerical preference				−0.06	0.07	−0.76	−0.05	0.08	−0.63
Empathic concern				−0.05	0.10	−0.50	−0.11	0.10	−1.07
Attitudes (nucl. energy)							−0.20	0.09	−2.13 *
Trust in authorities							−0.18	0.07	−2.49 **
	R^2^ = 0.011 F(5, 613) = 1.307			R^2^ = 0.011 F(7, 584) = 0.922			R^2^ = 0.032 F(9, 510) = 1.890 *		

*Note.* * = *p* < 0.05, ** = *p* < 0.01.

**Table 3:** Effect of message type on message acceptance.

Effect of Message Type on Message Acceptance									
	Model 1			Model 2			Model 3		
Predictor	β	SE	T	β	SE	t	β	SE	T
(Constant)	−2.71	5.46	−0.50	4.30	5.85	0.74	4.17	6.40	0.65
*Message type* *(reference category: Numerical)*									
Narrative (dummy)	−0.40	0.11	−3.54 ***	−0.39	0.12	−3.40 **	−0.47	0.12	−3.90 ***
Combined (dummy)	−0.26	0.11	−2.32 *	−0.27	0.12	−2.36 *	−0.29	0.12	−2.36 *
*Control variables*									
Gender	−0.20	0.09	−2.08 *	−0.10	0.10	−1.01	−0.04	0.11	−0.39
Age	0.00	0.00	1.17	0.00	0.00	−0.09	0.00	0.00	0.29
Education	−0.01	0.02	−0.25	0.00	0.02	−0.01	0.00	0.02	0.13
Numerical preference				0.08	0.05	1.60	0.08	0.05	1.55
Empathic concern				−0.12	0.07	−1.77	−0.07	0.07	−1.03
Attitudes (nucl. energy)							0.20	0.06	3.21 ***
Trust in authorities							0.22	0.05	4.43 ***
	R^2^ = 0.032 F(5, 584) = 3.898 **			R^2^ = 0.042 F(7, 556) = 3.446 **			R^2^ = 0.12 F(10, 490) = 6.420 ***		

*Note.* * = *p* < 0.05, ** = *p* < 0.01, *** = *p* < 0.001.

**Table 4:** Effect size by dependent variable and group comparison.

DV	Group Comparison	Cohen’s d
*Message credibility*	Numerical - Narrative	0.27
	Numerical - Combined	0.09
	Narrative - Combined	0.19
*Message bias*	Numerical - Narrative	0.04
	Numerical - Combined	0.02
	Narrative - Combined	0.02
*Message acceptance*	Numerical - Narrative	0.36
	Numerical - Combined	0.23
	Narrative - Combined	0.13

**Figure A2:**
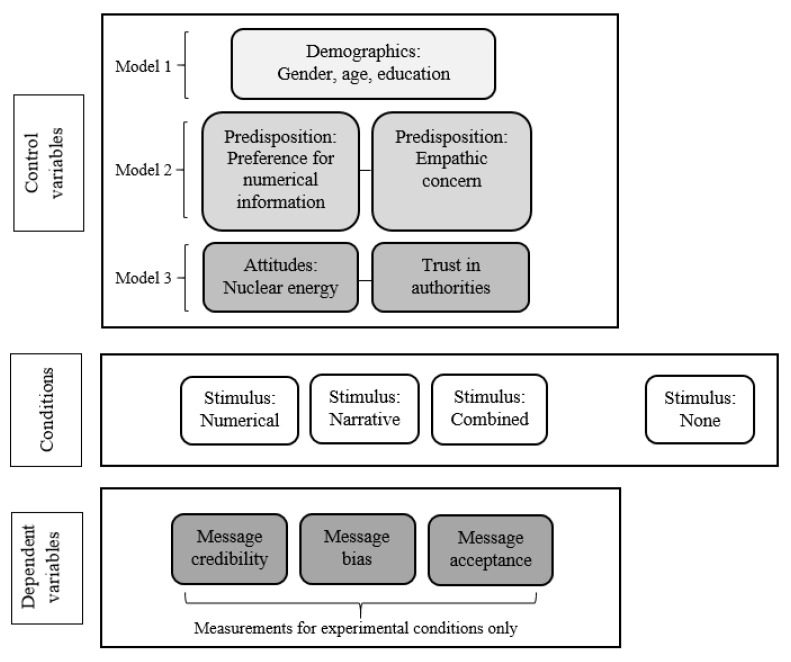
Experiment design.

## Appendix A

**Table A1:** Mean differences in risk perception between experimental and control groups.

	Mean Agreement		Effect Size (Cohen’s d)
	Experimental Groups (*N* = 614)	Control Group (*N* = 458)	
Statement 1: “If I needed a specific ingredient, I would buy it even if it was produced in Fukushima: to what extent do you agree or disagree?”	2.34 (SD: 1.26)	1.73 (SD: 1.07)	0.52
Statement 2: “I would feel uncomfortable eating food produced in Fukushima: to what extent do you agree or disagree?”	3.65 (SD: 1.17)	4.33 (SD: 1.04)	0.61

*Note*: Measured on 5-point agreement scale, higher levels indicate stronger agreement.

**Table A2:** Descriptive values per dependent variable, experimental condition.

DV	*N*	Mean	Std. Dev.
Credibility			
*Numerical*	220	3.64	1.64
*Narrative*	200	4.10	1.80
*Combined*	199	3.78	1.50
Bias			
*Numerical*	220	4.00	1.65
*Narrative*	200	3.94	1.78
*Combined*	199	3.97	1.56
Acceptance			
*Numerical*	211	3.40	1.10
*Narrative*	189	2.99	1.20
*Combined*	190	3.14	1.12
